# Assessment of Silicone Particle Migration Among Women Undergoing Removal or Revision of Silicone Breast Implants in the Netherlands

**DOI:** 10.1001/jamanetworkopen.2021.25381

**Published:** 2021-09-20

**Authors:** Henry B. P. M. Dijkman, Inca Slaats, Peter Bult

**Affiliations:** 1 HAN University of Applied Sciences, Institute of Applied Biosciences and Chemistry, Nijmegen, the Netherlands; 2 Department of Pathology, Radboud University Medical Center, Nijmegen, the Netherlands

## Abstract

**Importance:**

Silicone breast implants have been on the market for breast augmentation or breast reconstruction for approximately 60 years but may lead to medical complications, also called *breast implant illness*.

**Objective:**

To evaluate the existence of silicone gel bleed and migration over a long time period, including the period in which the newer cohesive silicone gel breast implants were used.

**Design, Setting, and Participants:**

In this single-center case series, capsule tissue and lymph node samples were collected from women who underwent removal or revision of silicone breast implants from January 1, 1986, to August 18, 2020, and data were extracted from the pathological reports and revision of the histology if data were missing. All tissues were examined using standard light microscopy, some extended with modified oil red O staining and energy-dispersive radiographic spectroscopy. A total of 365 women had capsular tissue removed, including 15 patients who also had lymph nodes removed, and 24 women had only lymph nodes removed. Data were analyzed from January to May 2021.

**Exposures:**

Silicone breast implants.

**Main Outcomes and Measures:**

The main outcome was presence or absence of silicones inside or outside the capsule. One-way analysis of variance was used to determine significance between groups.

**Results:**

Among a total of 389 women with silicone breast implants (mean [SD] age, 50.5 [11.2] years), 384 women (98.8%) had silicone particles present in the tissues, indicating silicone gel bleed. In 337 women (86.6%), silicone particles were observed outside the capsule (ie, in tissues surrounding the capsule and/or lymph nodes), indicating silicone migration. In 47 women (12.1%), silicone particles were only present within the capsule. In 5 women (1.2%), no silicone particles were detected in the tissues. Patients were divided into 2 groups, with 46 women who received cohesive silicone gel breast implants and 343 women who received either an older or a newer type of breast implant. There were no differences in silicone gel bleed or migration between groups (silicone detected outside or inside capsule: 44 women [95.7%] vs 340 women [99.1%]; *P* = .19).

**Conclusions and Relevance:**

In this case series including women with noncohesive or cohesive silicone gel breast implants, silicone leakage occurred in 98.8% of women, indicating silicone gel bleed, and in 86.6% of women, migration of silicone particles outside the capsule was detected.

## Key Points

**Question:**

Are cohesive gel breast implants associated with silicone bleed?

**Findings:**

In this case series including 389 women with silicone gel breast implants, 384 women (98.8%) showed silicones in the tissues. There was no statistically significant difference between women with cohesive gel implants and those with noncohesive gel implants.

**Meaning:**

These findings suggest that cohesive silicone gel breast implants leak silicones and may potentially harm patients, as noncohesive gel implants do.

## Teaser

This case series examines the presence of silicone bleed and migration among women with cohesive or noncohesive gel silicone breast implants.

## Introduction

Silicone breast implants (SBIs), first marketed in 1962, are used worldwide for reconstructive and cosmetic purposes. Approximately 25 000 people receive SBIs in the Netherlands annually, compared with approximately 400 000 people in the United States, and millions of people around the world. In the past decades, there has been an ongoing discussion about the toxicity of SBIs, and an association with diseases and complications has been suggested.^1,2^

Recently, attention has refocused on SBI-associated anaplastic large cell lymphoma (BIA-ALCL).^3,4^ The major 2019 recall of specific SBIs owing to associations with BIA-ALCL, of which incidence is rising, has highlighted safety concerns with cosmetic SBIs and the increasing number of patients with breast implant illness.^5,6,7,8,9^ Breast implant illness is used to describe various complications associated with SBIs, ranging from brain fog, hair loss, fatigue, chest pain, sleep disturbances, irritable bowel syndrome, headaches, chronic pain all over the body, and autoimmune diseases, such as lupus and fibromyalgia.^8^

Silicones can trigger an immune response. In addition, enzyme biodegradation and hydrolyzation degrades silicone gel to cyclic silicones, leading to toxic effects and rupture.^10,11,12,13^ As early as 1995, Brautbar et al^14,15^ proposed that there was evidence for a causal relationship between implants and disease.

SBIs bleeding of small quantities of silicone gel has previously been associated with a higher capsule formation, which is assessed with the Baker score.^16^ When an SBI is implanted in the body, the body will react with capsule formation. It is generally known that the noncohesive SBIs show silicone gel bleed, because the silicone is fluid. This means that silicone implants sweat, releasing silicone particles, and these particles can end up inside the capsule or migrate into the surrounding tissue and in the body.^17^

An increase in the amount of silicones in tissues is often associated with increased numbers of macrophages, fibroblasts, giant cells, and contractile myofibroblasts,^18,19^ and this increase is associated with several harms and complications.^9^ Also, the surface of SBIs can affect the immune system.^20^

Data on silicone leakage from cohesive gel SBIs is scarce,^21,22^ and little data exist concerning leakage and migration. A recent case of silicone and sarcoid granulomas in a patient with highly cohesive SBIs was described in a 2021 study by Nanayakkara et al.^23^

Large case series in which data on gel bleed and migration in patients with noncohesive and cohesive gel SBIs are compared are lacking. The aim of this study is to gain more clarity and insights about the prevalence of silicone leakage in patients with noncohesive or cohesive SBIs.

## Methods

The Dutch national ethical guidelines state that no ethical approval is required for the use of anonymous leftover tissue, and this is also part of the standard treatment agreement in the Radboud University Medical Center.^24^ Therefore, this case series was exempt from ethical review board review and informed consent. None of the patients from whom leftover tissue was used refused the use of leftover tissue. This report follows the reporting guideline for case series.

### Patients and Tissues

In this case series, capsules and lymph nodes (when excised) were investigated from women undergoing explantation or revision surgery between January 1, 1986, and August 18, 2020, in the Netherlands. The patients received their SBIs in various clinics within the Netherlands.

Using the local pathology database of the Radboud University Medical Center, 2 search strategies were combined (one using the term *silicone* and one using the term *breast*) to retrospectively select all cases related to SBIs from January 1, 1986, to November 11, 2019. Women with implant-related complications from November 11, 2019, and August 18, 2020, were also prospectively included. All tissue samples were fixed in 4% formaldehyde, routinely processed, and embedded in paraffin.

### Histology

All tissue samples were analyzed with standard hematoxylin and eosin (HE) staining. In selected samples, an additional modified oil red O (MORO) staining was performed or an energy-dispersive radiographic spectroscopy (EDX) analysis was done.

In HE staining, silicones can be recognized as glassy, nonbirefringent droplets in vacuoles in the tissue or intracellular in macrophages. This appearance is unique for silicones and is not found in controls without SBIs. Also, the presence of brown or black granules in macrophages may be an indicator of the presence of silicone particles. These vacuoles and the presence of granules are extensively investigated and confirmed for silicones with the 3-phase technique, a combination of HE staining, MORO staining, and EDX analysis described in gel bleed and rupture of SBIs.^17^

### Statistical Analysis

All samples and databases were anonymized before use. From the selected samples, the anonymized pathological reports were analyzed. If data on histological analysis were missing concerning the presence of silicones, the microscopical slides were reviewed (by I.S. and, if in doubt about the diagnosis, also by P.B.) and the following data were collected: age, incidence year, clinical data, conclusion, diagnosis code, reason for augmentation, side, location, presence of silicones in the capsule, presence of silicones outside the capsule, presence of histiocytic or inflammatory response, staining and technique (HE, MORO, or EDX), presence of silicones in lymph nodes (if excised), presence of BIA-ALCL or other malignant neoplasm. For women with a double-sided capsule resection (left and right), the side that showed the most extensive silicone migration (silicones outside the capsule) was used in our study. Also, for women who had a capsule resection more than once, the sample that showed the most extensive silicone migration at the youngest age was used in our study.

As use of the newer SBIs with cohesive silicone gel was beginning in 1995 in the Netherlands, we divided our study group in 2 groups: one group of women with cohesive gel SBIs in whom the histological examination was performed after the year 1999, and one group of all other women, including both older noncohesive SBIs or newer SBIs. Since we did not have full data on brand or type of SBI, all women for whom we were unsure of type of SBI were included in the second group.

We used 1-way analysis of variance statistics to determine statistically significant differences of silicone migration between groups. Statistical analyses were performed using SPSS statistical software version 27 (IBM). *P* = .05 was considered significant. Data were analyzed from January to May 2021.

## Results

A total of 389 women with SBIs (mean [SD] age, 50.5 [11.2] years) were included. Based on the 2 searches in the pathology database of the Department of Pathology of the Radboud University Medical Center, a total of 365 women with resected capsules surrounding SBIs were included in this study (including 15 women with excised lymph nodes). A total of 6 women had BIA-ALCL, of whom 5 women did not have a capsule resection in our pathology database; these 5 women underwent operations in other hospitals and were not included in the study population analyzed. In 41 women, lymph nodes were resected, including 24 women without resected capsules.

Because the clinical information given in orders for histopathological examination was very limited, we are not able to provide reliable data on brand of SBIs, reasons for capsule removal, or patient concerns. However, we divided our study group into 2 groups. The first group included 46 women (11.8%) who received cohesive gel SBIs. This group includes 31 women with confirmed clinical data for cohesive gel SBIs and 15 women with corresponding age at histological examination for cohesive gel implants (year of histological examination: 2000-2020 and age at histological examination: ≤20 years or 21-40 years) (Table 1). The second group included 343 women (88.2%) who received either a noncohesive gel SBI or a newer type of SBI. The histological data were reviewed in 307 women (78.9%) because of insufficient information about the presence and location of silicones given in the pathological report.

**Table 1.** Overview of Results of Silicone Presence Inside and Outside the Capsule, Including Lymph Nodes, and Presence of ALCL

Characteristic	No. (%) (N = 389)
Resected capsules	365 (93.8)
Only lymph nodes examined	24 (6.2)
Silicones in tissues surrounding the capsule or in lymph nodes^a^	337 (86.6)
Silicones inside capsule only	47 (12.1)
No silicones detected	5 (1.3)
Cohesive silicone gel breast implants^b^	46 (11.8)
Silicones outside capsule	38 (82.6)
Silicones inside capsule only	6 (13.0)
No silicones detected	2 (4.3)
Lymph nodes examined	10 (21.7)
Silicones in lymph nodes	10 (100)
Noncohesive silicone gel or unknown type of breast implants	343 (88.2)
Silicones outside capsule or in lymph nodes	299 (87.2)
Silicones inside capsule only	41 (12.0)
No silicones detected	3 (0.9)
Lymph nodes examined^a^	31 (9.0)
Silicones in lymph nodes	30 (96.8)
No silicones in lymph nodes	1 (3.2)
ALCL	6 (1.5)
Silicones outside capsule	1 (16.7)

Abbreviation: ALCL, anaplastic large cell lymphoma.

^a^ Including 24 women with only lymph nodes examined.

^b^ Including 31 women with confirmed clinical data for cohesive gel breast implant, with 15 cohesive gel implants determined based on age and year of histological report.

The results of silicone presence inside and outside the capsule and in resected lymph nodes are summarized in Table 1. Silicones limited to the capsule were found in 47 women (12.1%), whereas in 337 women (86.6%), silicones were detected in tissues surrounding the capsule and/or in lymph nodes. Therefore, a total of 384 women (98.8%) had silicones detected in the tissue resections. The Figure presents a woman with a high cohesive SBI with extensive silicone depositions in the tissues and histiocytic reaction. In only 5 women (1.2%), no silicones could be found in the tissues, neither inside nor outside the capsule. In only 1 women with ALCL, a capsule resection was examined in our laboratory, and silicones were detected outside the capsule. Of 41 women with resected lymph nodes, 40 (97.6%) had silicones within the lymph nodes, including 10 women (24.4%) with resected lymph nodes in the group with cohesive gel. All 10 of these women had silicones within the lymph nodes. Of 46 patients with cohesive gel SBIs, 2 (4.3%) did not have silicones inside or outside the resected capsules (Table 1). In a total of 93 samples (23.9%), MORO staining was performed, confirming the presence of silicones, and in 6 samples (1.5%), EDX analysis was conducted, which also confirmed the presence of silicones (Table 2).

**Figure.**
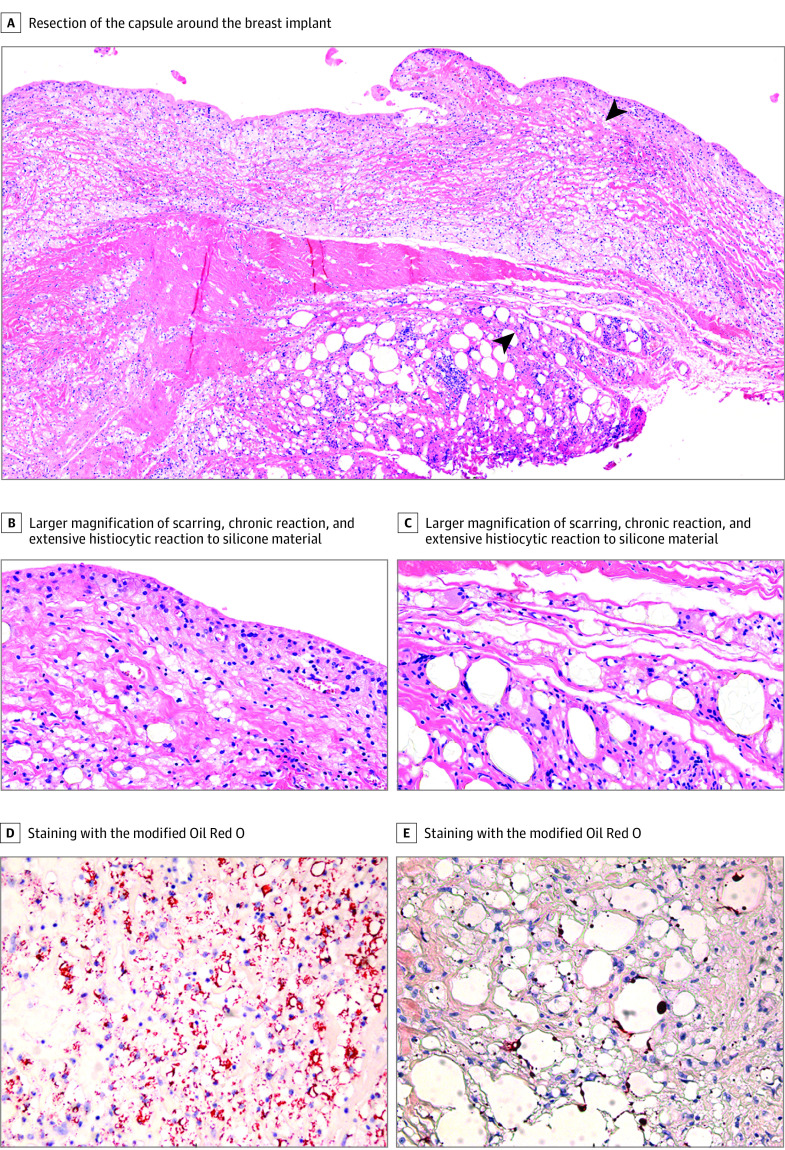
Silicone Migration in a Patient With a High Cohesive Gel Implant A-C illustrate a 4-μm paraffin slide with standard light microscopy. A, Scarring, chronic reaction, and extensive histiocytic reaction to silicone material (arrows), with larger magnification in panels B and C. D and E, Silicone fragments are found locally between striated muscle cells outside the capsule. Microscopic magnification: A, 100×; B, C, D, and E; 400×.

**Table 2.** Results of Additional MORO Staining and EDX Analysis

Silicones detected	Women, No.	No. (%)	
		Confirmed with MORO	Confirmed with EDX
In tissues surrounding the capsule or in lymph nodes	337	87 (25.8)	6 (1.5)
Inside capsule only	47	6 (12.8)	Not performed

Abbreviations: EDX, energy-dispersive radiographic spectroscopy; MORO, modified Oil Red O.

The frequency of histiocytic reaction or inflammatory infiltrate is summarized in Table 3. In 360 women (92.5%), a histiocytic response or inflammatory infiltrate within or outside the capsule was present. In only 4 women (1.0%), no histiocytic or inflammatory infiltrate was observed. Information about inflammation in the pathology report was missing for 25 women (6.4%).

**Table 3.** Presence of Histiocytic Reaction or Inflammatory Infiltrate in the Tissue Resections

Silicones detected	Women, No. (N = 389)	No. (%)		
		Histiocytic reaction or inflammatory infiltrate present (n = 360)	No histiocytic reaction/inflammatory infiltrate present (n = 4)	Unknown (n = 25)
In tissues surrounding the capsule and/or in lymph nodes	337	316 (93.8)	2 (0.6)	19 (5.6)
Inside the capsule only	47	40 (85.1)	1 (2.1)	6 (12.8)
None	5	4 (80.0)	1 (20.0)	0

To look for potential differences in silicone spread associated with age, the patients were divided in 4 age groups: younger than 30 years, 30 to 44 years, 45 to 59 years, and 60 years or older. For each age group, we determined the presence and location of silicones (Table 4). There were no differences in presence of silicones outside the capsule or inside the capsule by age group (Table 4).

**Table 4.** Silicone Presence Inside and Outside the Capsule Divided by Age Groups

Silicones detected	No. (N = 389)	Age group, No. (%), y				
		<30 (n = 10)	30-44 (n = 103)	45-59 (n = 201)	≥60 (n = 73)	Unknown (n = 2)
In tissues surrounding the capsule and/or in lymph nodes	337	8 (2.4)	88 (26.1)	173 (51.3)	66 (19.6)	2 (0.6)
Inside the capsule only	47	1 (2.1)	13 (27.7)	27 (57.4)	6 (12.8)	0
None	5	1 (20.0)	2 (40.0)	1 (20.0)	1 (20.0)	0

The silicone spread was compared between women who received cohesive gel SBIs and women who received either a noncohesive gel or a newer SBI. There was no statistically significant difference between the groups in respect to the presence of silicones inside or outside the capsule (44 women [95.7%] vs 340 women [99.1%]; *P* = .19).

## Discussion

In this case series, we found that most women (98.8%) with SBIs had silicone depositions in the tissues examined and that in most women (86.6%), the silicones were also found outside the capsule (ie, in the surrounding connective, fat, or muscle tissue) and/or in lymph nodes, irrespective of type or brand of SBI, including noncohesive or cohesive silicone gel. Aside from the fact that all women had capsule formation around the breast implant, in most women (92.5%), an inflammatory reaction (eg, macrophages with phagocytosed silicones) was observed, which is also associated with silicone leakage.

In 1995, Brautbar et al^15^ proposed that silicones were not inert, could induce cell death, and had mutagenic effects. In essence, Brautbar et al^15^ concluded that breast implant devices were delivery systems of silicone molecules, not inert and unrelated to rupture, because their contents were mixtures of various-sized polymer compounds, smaller than the pores of the shell, and bleeding occurred. Still, debate exists about the causative relationship between SBIs and breast implant illness,^1^ although many researchers recognize it as a fact.^2^ The finding that explantation of SBIs was associated with a considerable decline in symptoms in many women who were considered to have complications of their SBIs is an indicator of a potential causative relationship. A 2017 study by Colaris et al^2^ found a reduction in symptoms in 50% of women with SBIs after explantation, and a 2020 study by Wee et al^25^ reported a significant and sustained (beyond 30 days) improvement in 11 common symptom domains. A 2020 study by Kappel and Pruijn^26^ also found a significant reduction of symptoms in their patient group after explantation. Another study by Wee et al^27^ found that patients presenting with symptomatic SBIs with pulmonary symptoms had significant improvement in pulmonary function after complete implant and capsule explantation.

Although we found silicones in the tissues examined in 98.8% of women with SBIs, the silicone particles were to a lesser extent also found outside the capsule. We can give some possible explanations for this. In some of the available microscopical slides, only a small piece of tissue from the total capsule was present, and not all capsules contained surrounding tissue, which might have led to sampling error. Furthermore, most samples were examined with HE only. In our experience, MORO staining is more sensitive to detecting silicones. Therefore, we believe that our results may even underestimate the true migration of silicones.

In our study, we included 389 women over a long period of time (January 1986 to August 2020). At least 46 women (11.8%) received cohesive SBIs. We did not see differences in silicone gel bleed or migration between women who received the newer cohesive SBIs and those who received noncohesive SBIs.

The amount of gel bleeding has already been described to correlate with capsular contraction,^28,29,30^ but less is known about migration into the body. However, we have reported in a 2016 study^17^ that silicone molecules can migrate to every spot in the body. In this study, lymph nodes were also resected in 41 women, and in 40 women (97.6%), silicones were observed in these lymph nodes, further supporting the migration of silicones into the body.

Although malignant neoplasms associated with SBIs are reported rarely, BIA-ALCL has become increasingly common over the years, and the incidence of this form of cancer has therefore been included in our study. We found that BIA-ALCL was present in 6 of 394 women (1.5%). Natural killer cell activity is known to decrease significantly in women with SBIs, thereby inhibiting the clearing of the silicones, and it is also thought that patients with decreased natural killer cell activity have an increased risk of cancer.^31^ In a case report,^32^ a patient aged 46 years was diagnosed with BIA-ALCL 2 years after her implants were removed. The fact that the lymphoma developed 2 years after the removal of the implants and the cancer occurred in the axillary lymph nodes suggests that migration of silicones to the lymph nodes can be a risk and must be taken seriously.^32^

BIA-ALCL is an emerging and important medical challenge. The pathogenesis of BIA-ALCL has not been fully established so far, and there are several theories, of which silicone toxicity is one. Depending on the migration pattern, concentration, and location of silicone and its degradation products, different symptoms can occur. For example, silicone migration to the lymph nodes can trigger an immune system response, whereas silicone migration to the nerves or spinal cord can cause immobility.^33,34^

In assessing different age groups within this study, we did not find an association of silicone migration with age, nor did we find a difference in gel bleed and migration of silicones between noncohesive and cohesive gel SBIs.

Silicones are foreign to the body, and when silicone particles enter the body, the immune system can be triggered and toxic effects can occur. The amount of silicones lost from an implant is correlated with severity of symptoms, so data regarding silicone migration into the body are valuable. As the potential harm of SBIs is recognized now, the US Food and Drug Administration has given information for how to communicate these potential harms with patients in their latest guidance for breast implants.^13^

### Limitations

This study has some limitations. Drawing conclusions about different brands of SBIs in regards to silicone migration is difficult in our study because we do not have all the data. However, we found that most SBIs, regardless of brand or type, bled and showed the same pathogenesis. The shell or outer layer of the implant, with or without texture, is still made of silicones. This study was largely retrospective. However, by using 2 search strategies in our pathology database (based on the nationwide pathology database in which all pathology data in the Netherlands are registered) we are confident that we included all potential patients. Owing to the fact that we did not have access to the clinical history of the patients, except for the information given in the pathological report, we were not able to correlate silicone bleed and migration to clinical symptoms. Furthermore, we did not have the information for most women on whether they received more than 1 implant, eg, a noncohesive implant in the 1980s and a cohesive implant in the 2000s.

## Conclusions

In this case series, we found that noncohesive as well as cohesive gel SBIs bleed silicones, resulting in migration of silicones beyond the capsule, which occurred in 86.6% of women investigated. As SBIs are associated with complications and even cancer, women should be well informed about the potential harms before these implants are placed into the body.

As the safety of SBIs is questioned more and more, we propose some recommendations. First, future studies should focus more on a potential causative relationship between silicones and complications and malignant neoplasms (BIA-ALCL), including samples from healthy women without SBIs and women with implants with and without signs of toxic effects. Such analyses might include blood analysis with ciRNaseq technology for early stage detection of silicone toxic effects, other blood analyses (eg, antibodies against silicones, cytokine levels, number of different types of inflammatory cells), analyses of hairs of the crown to detect systemic spread of silicones (eg, platinum levels), tissue analyses (eg, amount of silicones, type and amount of inflammatory cells, cytokine levels), and analysis of potential (eg, genetic) predisposition of women for developing silicone-related complications.

Second, the use of silicone gel–filled implants should be stopped until their safety has been proven. For breast reconstruction, autologous reconstruction should be used as often as possible. If silicone shell breast implants are used (when other options are not possible), they should be filled with saline or other known nontoxic fluids. If patients experience complications from their SBIs, explantation (with complete excision of the capsule) should be offered. The costs of this procedure should be reimbursed by the health insurance companies.
